# High-frequency, high-intensity electrical stimulation selectively activates human fast-spiking interneurons

**DOI:** 10.3389/fnsyn.2026.1843277

**Published:** 2026-06-26

**Authors:** Jaeyoung Yoon, Ricardo Silva, Scellig Stone, Hart Lidov, Emily Osterweil, Brielle R. Ferguson

**Affiliations:** 1 F. M. Kirby Neurobiology Center, Boston Children's Hospital, Boston, MA, United States; 2 Department of Neurology, Harvard Medical School, Boston, MA, United States; 3 Department of Neurobiology, Harvard Medical School, Boston, MA, United States; 4 Department of Neurosurgery, Boston Children's Hospital, Boston, MA, United States; 5 Department of Neurosurgery, Harvard Medical School, Boston, MA, United States; 6 Department of Pathology, Boston Children's Hospital, Boston, MA, United States; 7 Department of Pathology, Harvard Medical School, Boston, MA, United States; 8 Zander & Wyss Translational Neuroscience Center, Boston Children's Hospital, Boston, MA, United States; 9 Department of Genetics, Harvard Medical School, Boston, MA, United States

**Keywords:** cortical stimulation, electrical stimulation, epilepsy, excitation-inhibition balance, fast-spiking interneuron, human brain slice, parvalbumin interneuron, patch clamp electrophysiology

## Abstract

Electrical brain stimulation has been used to treat epilepsy with therapeutic success, but without complete understanding of its cellular mechanisms of seizure suppression. Using acute human brain slices obtained from pharmaco-resistant epilepsy patients, we delivered high-frequency, high-intensity extracellular electrical stimulation while recording from neocortical layer 2/3 pyramidal neurons (PNs), fast-spiking interneurons (FSINs), and non-fast-spiking interneurons (nFSINs). Electrical stimulation led to an increasingly selective activation of FSINs at higher stimulation frequencies, which consistently fired with high fidelity throughout prolonged stimulation periods while both PNs and nFSINs were suppressed rapidly. In addition, stimulation caused a long-term rebalancing of synaptic weights through selective and differential depression of synaptic inputs, resulting in an approximately twofold increase in the normalized excitatory-to-inhibitory postsynaptic conductance at FSINs while not affecting PNs, thereby providing support for FSIN activation toward increased inhibitory tone and network stabilization. These stimulation-induced effects were accompanied by only minimal changes in neuronal intrinsic excitability.

## Introduction

Pharmaco-resistant epilepsy, or intractable epilepsy, accounts for more than 20 % of all epilepsy patients and represents a major cause of morbidity (Kwan et al., 2011). Surgical resection can provide substantial seizure freedom for intractable focal epilepsy (Wiebe et al., 2001; Thijs et al., 2019), but it is not feasible in a large proportion of cases such as when the epileptogenic zone overlaps with the eloquent cortex or when seizure onset is multifocal (Rosenow and Lüders, 2001; Duncan et al., 2016). Brain stimulation has emerged as another therapeutic strategy, with chronic or responsive neurostimulation having successfully demonstrated sustained seizure reduction in focal epilepsy patients (Fisher and Velasco, 2014). These procedures can involve electrode implantation at or near the seizure onset zone, intended for modulation of cortical network activity via electrical stimulation (Morrell, 2011; Toprani and Durand, 2023). Stimulation parameters may vary across patients and protocols, but often consist of charge-balanced biphasic pulses with sub-millisecond pulse widths in high-frequency, high-intensity trains on the order of 10 to 100 Hz at milliampere range, adjusted to achieve therapeutic benefit while avoiding adverse stimulation-induced effects (Merrill et al., 2005; Cogan, 2008). The effectiveness of stimulation protocols depends critically on these parameters, yet their optimization remains limited due to a lack of mechanistic understanding of neurostimulation effects (Fisher and Velasco, 2014).

Central to epilepsy pathophysiology is the disruption of excitation-inhibition (E-I) balance. Convergent experimental and clinical evidence indicates that seizures arise from imbalanced network conditions that allow neuronal hyperactivity to be generated and propagated across neural circuits (Paz and Huguenard, 2015; Staley, 2015; Cho et al., 2024). Accordingly, previous studies have shown that brain stimulation of cortical or subcortical structures can be effective at terminating or preventing seizures by means such as modulation of synaptic transmission, recruitment of inhibitory interneurons, or disruption of network synchrony (Berényi et al., 2012; Krook-Magnuson et al., 2013; Paz et al., 2013). It has also been proposed that stimulation may induce long-term plastic changes in epileptogenic circuits that can gradually reduce seizure propensity over longer periods (Korai et al., 2021; Appelbaum et al., 2023; Khambhati et al., 2021). The cellular mechanisms through which electrical stimulation can modulate cortical E-I balance to suppress seizures are incompletely understood, yet this question was previously inaccessible due to the prevalence of relatively less invasive methods in human studies. Separately, a primary caveat of rodent seizure models is that they differ from the human condition in terms of cellular organization and anatomical scale. In addition, clinical stimulation protocols utilize large-scale stimulation, in contrast to the more controlled manipulations used in most experimental settings.

In this study, we sought to gain mechanistic insight into the antiepileptic effects of electrical brain stimulation by delivering macroscopic stimulation in acute human brain slices prepared from cortical tissue resected from patients diagnosed with intractable epilepsy. We found that human neocortical layer 2/3 (L2/3) fast-spiking interneurons (FSINs) were selectively activated with high fidelity throughout the duration of high-frequency, high-intensity stimulation, while pyramidal neurons (PNs) or non-fast-spiking interneurons (nFSINs) were suppressed by the same stimulation and failed to fire correspondingly to higher stimulation frequencies. In addition to the selective modulation of the firing rates of neuronal subpopulations during the stimulation epoch, stimulation produced a persistent modification of single synaptic strengths which led to increased net excitatory postsynaptic conductance at FSINs but not at PNs. On the other hand, little to no changes in the intrinsic neuronal excitability were introduced by stimulation. These findings indicate that high-frequency, high-intensity electrical stimulation can dynamically restore cortical E-I balance through selective activation of FSINs during prolonged stimulation, followed by changes in the synaptic weights that can collectively stabilize network activity by enhancing the inhibitory tone.

## Results

### Human fast-spiking interneurons are selectively activated by high-frequency stimulation

**Figure 1:**
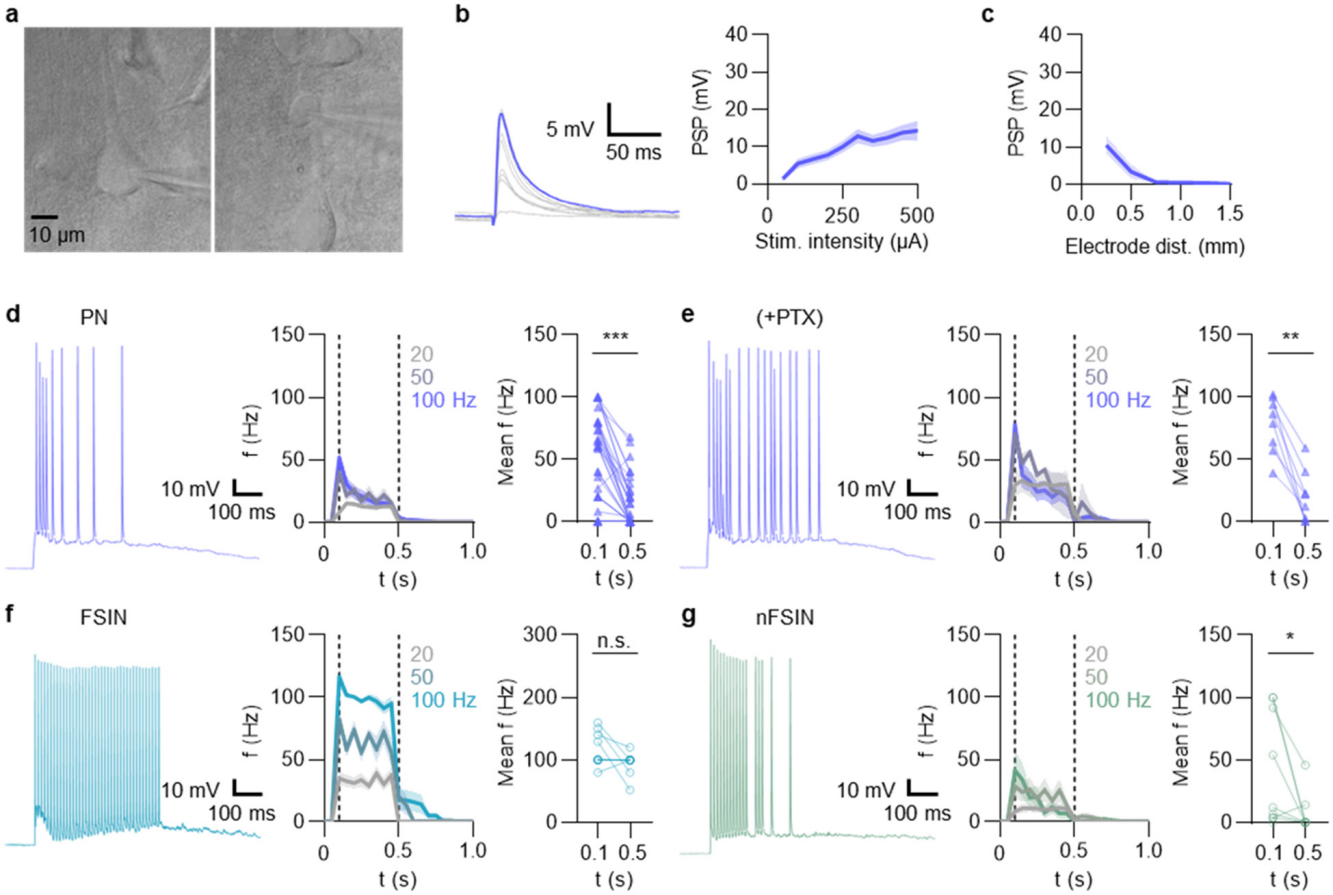
High-frequency, high-intensity electrical stimulation selectively activates human FSINs. **(a)** Representative pictures of a human L2/3 PN **(left)** and FSIN **(right)**. Slice quality could be inspected visually by tracing apical dendrites of PNs preserved up to the cortical surface without truncation (see Methods). **(b) (Left)** Representative traces from evoked PSPs with stimulation at 50 μA increments, with the highlighted trace corresponding to the largest stimulation intensity. **(Right)** PSP peak amplitude by stimulation intensity (*n* = 14 cells, 1 human). **(c)** PSP peak amplitude by distance between the cell and the stimulation electrode (*n* = 4 cells, 1 human). Suprathreshold responses at closer distances were not included. **(d) (Left)** Representative trace from 100 Hz stimulation; note that stimulation artifacts were removed from traces for clarity. **(Middle)** PN firing rates in response to stimulation. Dotted lines indicate the beginning and the end of stimulation trains (*n* = 25, 26, 33 cells, 5 humans; 20, 50, 100 Hz). PN firing rates in response to different stimulation frequencies were not significantly different (*P* = 0.1068, two-way ANOVA). **(Right)** PN firing rate was suppressed toward the end of 100 Hz stimulation (*P* < 0.0001, Wilcoxon signed-rank test). Mean firing rates were taken from the first and last 50-ms bins immediately after and before *t* = 0.1 and 0.5 s. **(e)** Same as **(d)**, but after bath application of 100 μM PTX (*n* = 2, 2, 8 cells, 2 humans). PN firing rates were similar across stimulation frequencies (*P* = 0.7479, two-way ANOVA), and suppressed with 100 Hz stimulation (*P* = 0.0078, Wilcoxon signed-rank test). **(f)** FSIN firing rates in response to stimulation (*n* = 8, 10, 10 cells, 5 humans). Prolonged stimulation resulted in the selective activation of FSINs which fired at increasingly higher firing rates at higher stimulation frequencies (*P* < 0.0001, two-way ANOVA), and with high fidelity without suppression (*P* = 0.1250, Wilcoxon signed-rank test), unlike PNs or nFSINs. Note different axis scales in **(f)**. **(g)** nFSIN firing rates in response to stimulation (*n* = 9, 8, 9 cells, 5 humans). nFSIN firing rates were not different across stimulation frequencies (*P* = 0.2640, two-way ANOVA), and suppressed toward the end of stimulation (*P* = 0.0391, Wilcoxon signed-rank test). ^*^P < 0.05, ^**^P < 0.01, ^***^P < 0.001; n.s., non-significant.

Human cortical neurons were recorded from acute brain slices obtained from patients between ages 1 to 21 (8.4 ± 2.0 years; Supplementary Table 1), who had resective surgery for intractable epilepsy (Figure 1a). For macroscopic stimulation, we delivered a train of 500 μA bipolar stimulation pulses at varying frequencies for a total duration of 400 ms (8, 20, 40 pulses respectively for 20, 50, 100 Hz). The stimulation intensity of 500 μA was chosen as a saturating condition to maximally activate nearby synaptic afferents (Figure 1b), at the electrode distance of ~250 μm laterally from the recorded cell (Figure 1c). Human L2/3 PNs responded reliably to 20 Hz stimulation, but not to stimulation frequencies generally exceeding their maximal intrinsic firing rates (Supplementary Figure 1), such that their firing rates were readily suppressed during 50 or 100 Hz stimulation to converge at similar levels compared to 20 Hz stimulation (Figure 1d). These effects were partly but not entirely mediated synaptically, as PNs were able to fire at relatively higher rates but still with qualitatively similar suppression in the presence of 100 μM A-type γ-aminobutyric acid receptor (GABA_A_R) antagonist picrotoxin (PTX) (Figure 1e). In contrast, FSINs fired continuously throughout the stimulation period without suppression at firing rates consistent with or even exceeding the stimulation frequency (Figure 1f). Such response was not common across all inhibitory interneuron subtypes, as nFSIN firing rates were suppressed similarly to PNs (Figure 1g). We note that our sample sizes were not sufficiently large to statistically quantify potential age-dependent effects within each cell type (Supplementary Figure 2), despite known developmental changes in neuronal properties including in humans (Barzó et al., 2025; Szegedi et al., 2024). These findings show that human FSINs selectively respond to high-frequency, high-intensity electrical stimulation with high firing fidelity, especially during prolonged (> 100 ms) stimulation.

**Figure 2:**
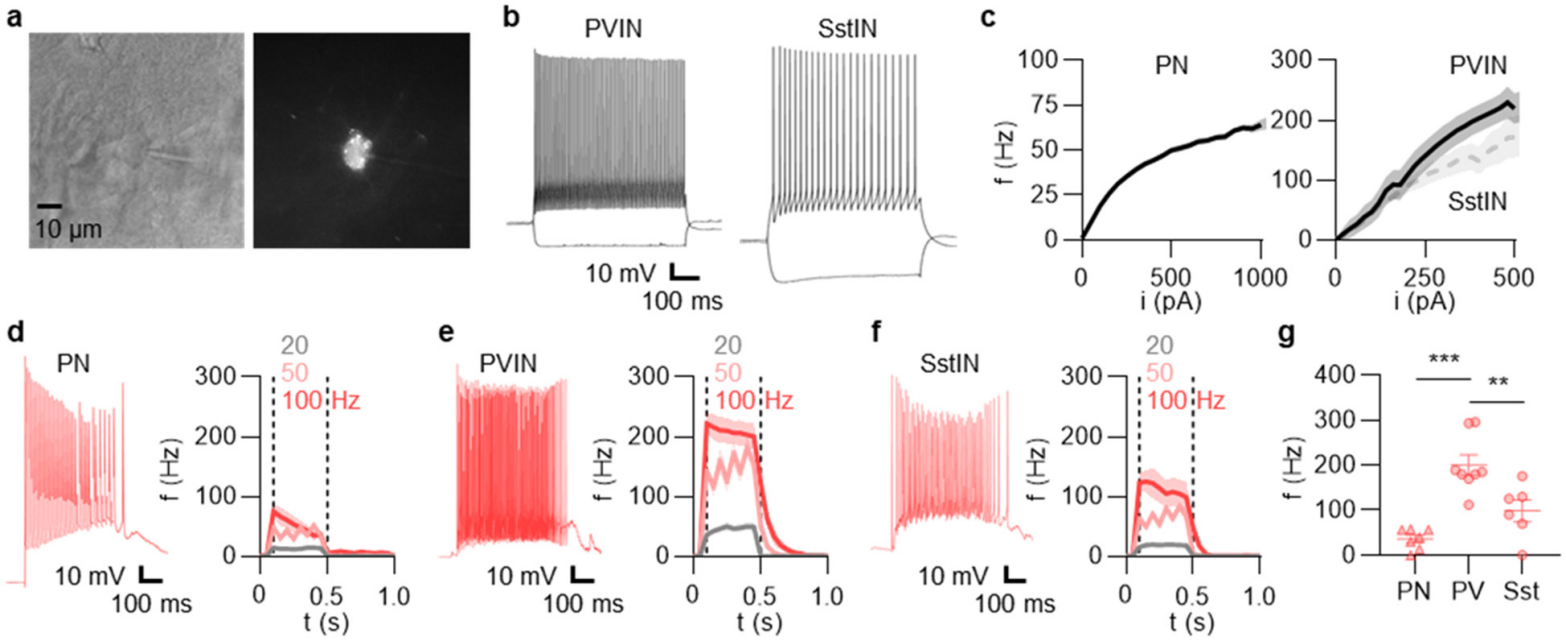
PVINs are selectively activated by stimulation. **(a)** Representative pictures of a virally labeled L2/3 PVIN (left, DIC image; right, mCherry expression at the patched cell). **(b)** Representative traces from a mouse PVIN and SstIN. Traces are from direct current injection at −200 pA and 2 × rheobase. **(c)** Intrinsic firing rates of mouse neuronal subtypes. **(Left)** PNs (*n* = 14). **(Right)** Solid lines, PVIN (*n* = 8); dashed lines, SstIN (*n* = 15). **(d)** Mouse PN firing rates in response to stimulation (*n* = 7, all frequencies). **(e)** Mouse PVIN firing rates in response to stimulation (*n* = 8). Similar to human FSINs, mouse PVINs were selectively activated by prolonged high-frequency stimulation trains with higher firing rates compared to mouse PNs or SstINs. **(f)** Mouse SstIN firing rates in response to stimulation (*n* = 6). **(g)** PVIN firing rates were higher than PN or SstIN firing rates. Firing rates were taken from the last 50-ms bins from each group, with 100 Hz stimulation (*P* = 0.0003, 0.0073; PN vs. PV, PV vs. Sst; Mann-Whitney *U* test). ^**^P < 0.01, ^***^P < 0.001.

Electrophysiologically defined FSINs represent a non-identical but highly overlapping population with transcriptomically defined parvalbumin-expressing interneurons (PVINs), whereas nFSINs are more weakly correlated with somatostatin-expressing interneurons (SstINs) amongst others (Markram et al., 2004; ; Hu et al., 2014; Tremblay et al., 2016). To determine whether the characteristic response of FSINs to stimulation was similarly observed in PVINs, we performed analogous experiments in the mouse prefrontal cortex using mCherry to virally label PVINs and SstINs (Figure 2a). All PVINs recorded had fast-spiking properties, while SstINs additionally displayed non-fast-spiking properties (Figure 2b). Mouse L2/3 PNs had higher overall intrinsic excitability compared to human PNs in terms of input resistance or rheobase (Supplementary Figure 3), and mouse PNs and interneurons exhibited higher firing rates compared to their human counterparts (Figure 2c). Regardless, mouse PN firing rates were also suppressed at higher stimulation frequencies that exceeded their average maximal intrinsic firing rates (Figure 2d), while mouse PVINs fired with high fidelity throughout the stimulation trains (Figure 2e). The difference between mouse PN and PVIN firing rates was more pronounced compared to human PNs and FSINs, as mouse PVINs often fired in doublets or more in response to a given stimulation pulse. While mouse SstIN firing rates were not as strongly suppressed as human nFSINs, they nevertheless fired at lower frequencies compared to PVINs (Figure 2f); we also note that, unlike PVINs, mouse SstINs represented a more electrophysiologically heterogeneous population as has been shown from previous studies, including fast-spiking SstINs (Supplementary Figure 4). PVINs were therefore selectively activated by stimulation over other neuronal populations (Figure 2g), similar to human FSINs.

### Cortical stimulation rebalances synaptic weights against network hyperactivity

**Figure 3:**
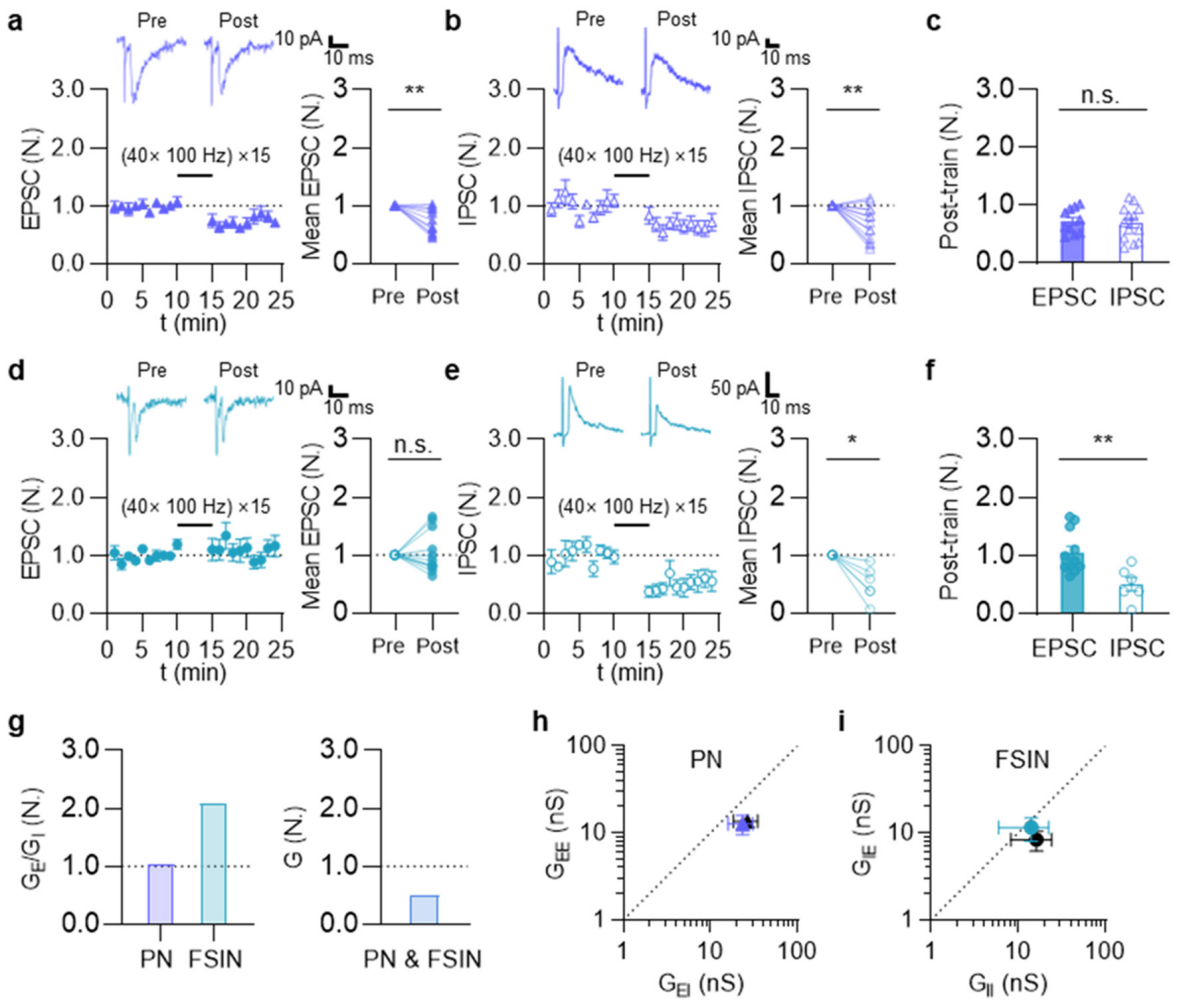
Redistribution of synaptic weights by stimulation. Minimal stimulation was delivered at the frequency of 0.1 Hz to test putative single synapses before and after the high-frequency, high-intensity stimulation trains. **(a)** Excitatory synapses onto postsynaptic human PNs were depressed following stimulation trains. **(Left)** EPSC vs. time, normalized to the average EPSC during baseline before stimulation (*n* = 10 cells, 3 humans). Representative traces are from minimally evoked responses before and after stimulation trains. **(Right)** Same data, averaged before and after baseline at each cell for pairwise comparison (*P* = 0.0039, Wilcoxon signed-rank test). **(b)** Inhibitory synapses onto postsynaptic PNs were depressed by stimulation (*n* = 13 cells, 1 human; *P* = 0.0046). **(c)** The amount of depression for excitatory and inhibitory synapses at postsynaptic PNs were not different (*P* = 0.6926, Mann-Whitney *U* test). Values are averaged post-train PSCs normalized to the respective baselines. **(d)** Excitatory synapses onto postsynaptic FSINs remained stable, unlike other synapse types (*n* = 11 cells, 3 humans; *P* = 1.0000, Wilcoxon signed-rank test). **(e)** Inhibitory synapses onto postsynaptic FSINs were strongly depressed, compared to other synapse types (*n* = 6 cells, 1 human; *P* = 0.0313). **(f)** Selective depression of inhibitory synapses onto postsynaptic FSINs by stimulation trains (*P* = 0.0048, Mann-Whitney *U* test). **(g) (Left)** Excitatory-to-inhibitory postsynaptic conductance at PNs and FSINs after stimulation trains, normalized to the respective ratios at baseline. **(Right)** Postsynaptic conductances after stimulation trains normalized to baseline, rearranged to represent synaptic excitability as a single variable G: = G_EE_G_II_/G_EI_G_IE_. G_XY_ denotes synaptic conductance from presynaptic population Y to postsynaptic population X, with E and I respectively representing excitatory and inhibitory population. **(h)** Excitatory vs. inhibitory postsynaptic conductance from saturating stimulation, at postsynaptic PNs (*n* = 3), before (black) and after (violet) high-frequency stimulation trains. **(i)** Excitatory vs. inhibitory postsynaptic conductance from saturating stimulation, at postsynaptic FSINs (*n* = 5), before (black) and after (cyan) high-frequency stimulation trains. ^*^P < 0.05, ^**^P < 0.01; n.s., non-significant.

We then examined whether high-frequency, high-intensity stimulation trains could introduce lasting changes in synaptic transmission via long-term potentiation (LTP) or long-term depression (LTD), in addition to the selective modulation of firing rates during stimulation. For this purpose, we delivered minimal stimulation to evoke excitatory or inhibitory postsynaptic currents (EPSCs or IPSCs, respectively) and compared them before and after the 500 μA, 100 Hz stimulation trains. For IPSC recordings, cells were first patched using a potassium-based internal solution for electrophysiological characterization of FSINs, then re-patched with a cesium-based internal solution to record IPSCs under voltage clamp at a depolarized holding potential corresponding to the reversal potential for non-selective cationic conductance through glutamatergic receptors (see Methods); similarly, EPSCs were recorded at the holding potential corresponding to the reversal potential for anionic conductance through GABA_A_Rs (see Methods). Synaptic blockers were not used to isolate EPSCs or IPSCs, as they would alter the stimulation condition. EPSCs at postsynaptic PNs were depressed after the high-frequency, high-intensity stimulation trains, to ~72 % compared to baseline before stimulation (Figure 3a). The depression of mean EPSCs may reflect changes in the synaptic success rate or the effectiveness of presynaptic activation by stimulation, as EPSC potency (failure-excluded mean) was not changed before and after stimulation (Supplementary Figure S5). Unexpectedly, IPSCs at PNs were also depressed, to ~69 % of baseline (Figure 3b); unlike EPSCs, however, depression of mean IPSCs was accompanied by a corresponding decrease in the potency. Excitatory and inhibitory synapses at postsynaptic PNs were therefore depressed to a quantitatively similar extent (Figure 3c), despite potential differences in their underlying mechanisms. In contrast, EPSCs at postsynaptic FSINs were neither depressed nor potentiated by the stimulation trains (Figure 3d), while IPSCs at FSINs were strongly depressed to ~50 % of baseline (Figure 3e), indicating input-dependent changes in the synaptic weights caused by stimulation (Figure 3f). We also note that the effects of stimulation trains on postsynaptic responses were mediated without changes in the kinetics of minimally evoked EPSCs or IPSCs (Supplementary Figure 5). Consequently, the net postsynaptic conductance at human PNs remained unchanged, while the excitatory-to-inhibitory postsynaptic conductance ratio at human FSINs more than doubled after the stimulation trains, resulting in lower overall synaptic excitability (Figure 3g).

A shift in the net postsynaptic conductance at human FSINs from excitation to inhibition during large-scale cortical activation has been characterized as a distinguishing feature of epilepsy (Cho et al., 2024). To test whether this property could be reversed by the high-frequency, high-intensity stimulation we used in the current study, we further compared the excitatory and inhibitory postsynaptic conductances from saturating stimulation, instead of minimal stimulation, before and after the stimulation trains. At postsynaptic PNs, stimulation was ineffective at producing changes in either excitatory or inhibitory postsynaptic conductance at saturating condition (Figure 3h). Similarly, no significant changes were found in the postsynaptic conductances at FSINs, in spite of small nominal changes toward larger excitatory and smaller inhibitory components (Figure 3i). These were somewhat unexpected given the selective depression of IPSCs but not EPSCs at postsynaptic FSINs in the previous experiments; nevertheless, it should be noted that IPSCs from saturating stimulation are not entirely monosynaptic, and thus would be jointly affected by not only the increased excitatory-to-inhibitory postsynaptic conductance ratio but also the decreased single inhibitory synaptic efficacy at postsynaptic FSINs.

### Cortical stimulation does not modulate neuronal firing rates outside of stimulation trains

**Figure 4:**
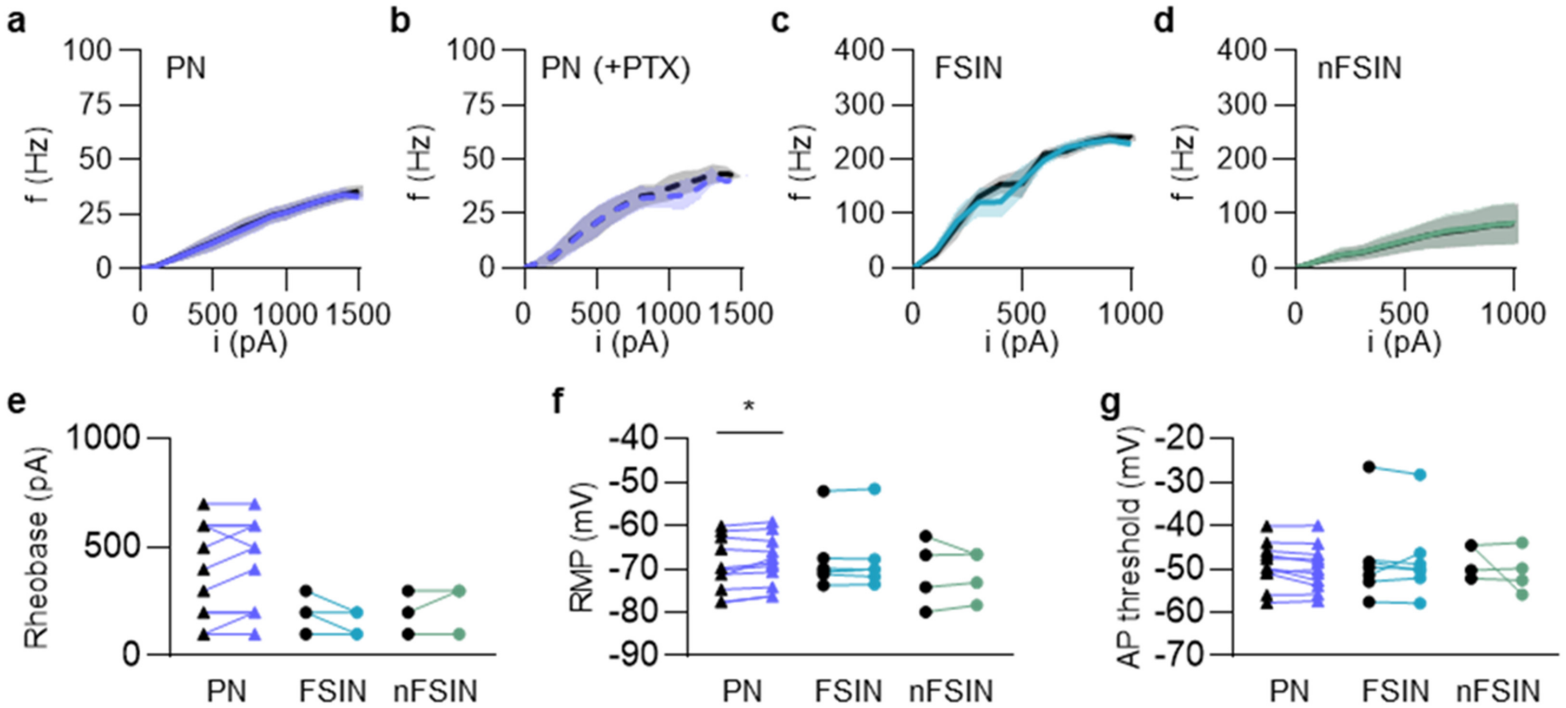
Intrinsic neuronal excitability is minimally affected by stimulation. **(a)** Human L2/3 PN firing rates, before (black) and (violet) after the 500 μA, 100 Hz stimulation trains (*n* = 11 cells, 1 human; *P* = 0.7932, two-way ANOVA). **(b)** Similar to **(a)**, but with 100 μM PTX (*n* = 4 cells, 1 human; *P* = 0.6426). **(c)** FSIN firing rates, before (black) and after (cyan) stimulation (*n* = 6 cells, 1 human; *P* = 0.8189). **(d)** nFSIN firing rates, before (black) and after (green) stimulation (*n* = 4 cells, 1 human; *P* = 0.9628). **(e)** Rheobase of neuronal subtypes. (*P* = 0.3750, 0.5000, 1.0000 for PN, FSIN, nFSIN; Wilcoxon signed-rank test) **(f)** PN RMP was significantly but minimally affected (−69.3 ± 1.9 vs. −68.4 ± 1.8 (mV); *P* = 0.0322), whereas FSIN and nFSIN RMP were not significantly changed (*P* = 0.5781, 0.8750). **(g)** AP threshold (*P* = 0.1289, 0.5781, 0.8750). ^*^P < 0.05.

In addition to synaptic weights, the firing rates of neuronal populations play a central role in shaping balanced cortical network states (Tsodyks et al., 1997; Hansel and Mato, 2013). Hence, we investigated whether the redistribution of synaptic weights by high-frequency, high-intensity stimulation was additionally accompanied by possible changes in the intrinsic excitability of excitatory and inhibitory neuronal populations, thereby affecting their firing rates outside of the stimulation epoch. However, human PN firing rates were not different before and after the stimulation trains (Figure 4a), neither with GABA_A_R block with PTX (Figure 4b). Likewise, FSIN firing rates were not affected (Figure 4c), nor were nFSINs firing rates (Figure 4d). Overall, stimulation produced only minimal changes in the neuronal intrinsic excitability. The rheobase of each population was not significantly different after stimulation (Figure 4e). PN resting membrane potential (RMP) was depolarized significantly, but only marginally (−69.3 ± 1.9 vs. −68.4 ± 1.8 (mV)), while FSIN and nFSIN RMP were unchanged (Figure 4f). Action potential (AP) threshold was also unchanged in all populations (Figure 4g). These results affirm that the long-term effects from the stimulation condition we tested were primarily mediated by synaptic mechanisms, without introducing meaningful changes in the intrinsic neuronal membrane properties.

## Discussion

Establishing a mechanistic framework that links clinical stimulation protocols to circuit-level modulation is critical to evidence-guided development of therapeutic stimulation strategies for epilepsy. Stimulation conditions in practice vary widely in terms of frequency, amplitude, pulse width, as well as electrode properties such as dimension and impedance; these parameters are in turn constrained by charge density limits at the electrode-tissue interface to prevent undesirable electrochemical reactions that can lead to neuronal injury or electrode deterioration, including water electrolysis, changes in pH, generation of reactive chemicals, or corrosion (Fisher and Velasco, 2014; Merrill et al., 2005; Cogan, 2008). Using a specific preset of parameters, we found that electrical stimulation can introduce selective changes in the firing rates of neuronal populations as well as the relative strengths of synaptic connections between them, which collectively supported enhanced activation of human FSINs.

FSINs and PVINs are highly overlapping inhibitory interneuron subtypes that are essential for normal cortical function, whose dysfunction and subsequent disruption of cortical E-I balance have been linked with several neurological disorders (Cho et al., 2024; Marín, 2012; Ferguson et al., 2018, 2023). In epilepsy, for example, decreased expression of PVINs or GABA_A_Rs on PNs have been found (Medici et al., 2016; Brooks-Kayal et al., 1998), and abnormal FSIN function has been implicated with the generation of pathological high-frequency oscillations (Jiruska et al., 2017). Here, we showed that human FSINs are selectively activated during high-frequency, high-intensity electrical stimulation due to their unique ability to fire in high fidelity throughout prolonged stimulation, whereas PN or nFSIN firing was readily suppressed by the same stimulation. In parallel experiments using viral labeling in mice, PVINs but not SstINs were similarly activated by high-frequency stimulation, indicating that the nFSIN or SstIN subtypes are less likely to mediate the stimulation-induced effects of network stabilization compared to FSINs or PVINs. It should be noted, however, that interneuron subtypes are not always mutually exclusive. For example, a smaller subset of interneurons has been identified to co-express PV and Sst, including in humans (Lee et al., 2023). Moreover, electrophysiologically defined nFSINs are known to comprise multiple subclasses, which include but are not limited to a subset of SstINs (Markram et al., 2004; The Petilla Interneuron Nomenclature Group (PING), 2008). The less effective recruitment of human nFSINs observed in our experiments hence likely reflects this heterogeneity, rather than representing a single uniform population.

In addition to acute effects on spiking, stimulation produced persistent modifications in the synaptic weights resulting in a selective change in the normalized excitatory-to-inhibitory conductance at postsynaptic FSINs toward their increased activation, without affecting PNs. Specifically, synaptic depression was found to different extents in all connection types except excitatory synapses onto postsynaptic FSINs, while inhibitory synapses onto postsynaptic FSINs were the most strongly depressed. Long-term plasticity has been characterized extensively in diverse contexts (Malenka and Bear, 2004; Castillo et al., 2011; Chiu et al., 2019; McFarlan et al., 2023), yet inhibitory long-term plasticity on postsynaptic GABAergic interneurons has not been explored until relatively recently (Sarihi et al., 2012). Moreover, we found synaptic depression using high-frequency stimulation, even though traditionally high-frequency stimulation or theta burst stimulation have been associated with LTP as opposed to LTD induction with low-frequency stimulation. Nevertheless, previous human studies support the presence of connection-specific plasticity rules, such as mGluR-dependent LTD of large postsynaptic responses from a single strong synapse or the coactivation of multiple weaker synapses (Szegedi et al., 2016). It should be noted, however, that we used macroscopic stimulation at saturating conditions in an attempt to mimic clinical neurostimulation conditions in slices, whereas typical LTP/LTD experiments use controlled synaptic activation at modest intensities to isolate the synapse in question. The mechanisms of inhibitory LTD at postsynaptic FSINs after high-frequency, high-intensity stimulation therefore requires further investigation in order to disambiguate its underlying factors.

Activity-dependent modulation of synaptic strength by short-term plasticity (STP) is another crucial determinant of cortical computation and E-I balance (Zucker and Regehr, 2002). Redistribution of the relative weights of excitatory and inhibitory inputs during continued neural activity can shape network behavior, which in turn supports cognitive function (Yoon et al., 2020). Despite their importance, STP characteristics at individual synapses caused by the selective modulation of neuronal firing rates during high-frequency stimulation could not be explored in the present study. Very little is known about synaptic plasticity mechanisms in the human cortex (Verhoog et al., 2013; Szegedi et al., 2024), particularly STP (Testa-Silva et al., 2014; Planert et al., 2025); moreover, previous literature has often used variable ionic conditions including notably extracellular Ca^2+^ concentrations higher than the physiological range (Lyckenvik et al., 2025), even though it is well known that these conditions non-linearly influence release probability and subsequently STP dynamics. Further human studies could therefore benefit from direct measurements of synaptic dynamics using paired patch-clamp recordings between identified neurons under physiological conditions for more precise quantification of STP as well as their modulation in a connection-specific manner.

Given the role of cortical FSINs or PVINs as the primary providers of inhibitory input to PNs (Pfeffer et al., 2013), their preferential recruitment during and after stimulation represents a plausible mechanism for suppressing network hyperexcitability and restoring E-I balance (Isaacson and Scanziani, 2011). The observation that these effects can be mediated by relatively simple means as non-selective electrical stimulation owing to the organization of the human cortical circuit is both unexpected and encouraging, which motivates additional experiments aimed at targeting these interneuron populations more effectively for improved therapeutic benefit. Based on these early results, we hypothesize that the general effectiveness of electrical stimulation protocols could be similarly characterized by exploring the parameter space for quantitative changes in the firing rates and synaptic weights of neuronal populations, particularly those with respect to FSINs. We expect that the integration of these measurements with systematic exploration of stimulation parameter space would contribute to the development of predictive, mechanistically grounded models of electrical neuromodulation for epilepsy treatment.

The use of human brain tissue raises some specific considerations in interpreting the findings of this study. First is the extent to which the specific circuit features of the human neocortex contribute to the selective recruitment of FSINs in response to stimulation. The human neocortex contains a higher proportion of inhibitory interneurons compared to rodents (Marín, 2025), with strong excitatory synapses onto GABAergic interneurons, particularly the basket cells and chandelier cells (Molnár et al., 2008). These properties are expected to favor efficient recruitment of FSINs upon synchronous, high-frequency activation to effectively suppress PN output. In addition, most of the human tissue used in this study were collected from pediatric patients. The electrophysiological properties of human neurons change across developmental stage, which is especially notable for infants (Szegedi et al., 2024; Barzó et al., 2025). The sample sizes in our experiments were not sufficiently large for a quantitative analysis of potential age-related effects of large-scale stimulation on network responses. Lastly, we made use of cortical tissue obtained from epilepsy patients, as our aim was to determine the effects of electrical stimulation used to treat epilepsy. Epilepsy is known to be associated with synaptic and circuit-level reorganization, including alterations in inhibitory function and E-I balance (Cohen et al., 2002; Tóth et al., 2017); generalization of our findings to the non-pathological cortex hence must be proceeded with caution. While analogous experiments from the mouse cortex suggests against a purely pathology-driven effect, future work using non-epileptic human tissue or complementary *in vivo* approaches would be beneficial to distinguish potential pathology-specific adaptations from general cortical mechanisms.

## Methods

### Human brain slice preparation

All protocols of this study were approved by the Boston Children's Hospital (BCH) Institutional Biosafety Committee (IBC) (A00001785), with the human experiment component approved additionally by the Institutional Review Board (IRB) (09-02-0043). All human participants gave informed consent without compensation. Neocortical tissue was surgically resected from the frontal, temporal, and parietal areas of the cortex, from male and female patients with pharmaco-resistant epilepsy (Supplementary Table 1); no grouping was made based on patient sex due to sample size.

Acute human brain slices were prepared similarly to the methods described in detail previously (Cho et al., 2024; Yoon, 2024, 2025). Briefly, resected tissue was immediately submerged in ice-cold, temperature-controlled cutting solution and transported to the laboratory within BCH. Prior to slicing, the tissue block was prepared to preserve cortical anatomy and maximize slice yield; detailed descriptions of the procedure including a video are available online (Yoon, 2025). Tissue was sliced on a vibratome (Leica VT1200S) with an orienting specimen disc (Leica 14048142068). Extra tissue and slices were donated to other research groups in BCH with the respective institutional approvals. 300-μm-thick slices were prepared, allowed to recover at ~36 °C for ~1 h in artificial cerebrospinal fluid (aCSF), then maintained at room temperature until the experiments. Slices were kept on cell strainers (pore size 100 μm) in a sealed container, with the aCSF replaced every ~6–12 h. The same aCSF was used throughout slice recovery, incubation, and recording, as no specialized recovery or incubation solution was necessary to maintain optimal slice quality and longevity. Slices remained healthy for typically ~48–72 h post resection, or longer.

### Slice electrophysiology

Patch clamp recordings were made in aCSF at ~36 °C. Bath temperature was maintained with an inline temperature controller (Scientifica), with aCSF perfused at a rate of ~1.7 mL/min using a peristaltic pump (Gilson Minipuls 3). The recording solution contained (in mM): 125 NaCl, 25 NaHCO_3_, 3 KCl, 1.25 NaH_2_PO_4_, 10 D-glucose, 1 sodium ascorbate, 3 sodium pyruvate, 1.2 MgCl_2_, 1.2 CaCl_2_, pH ~7.3, ~305 mOsm. The cutting solution contained (in mM): 165 sucrose, 20 HEPES, 25 NaHCO_3_, 2.5 KCl, 1.25 NaH_2_PO_4_, 20 D-glucose, 5 sodium ascorbate, 3 sodium pyruvate, 7 MgCl_2_, 0.5 CaCl_2_, pH adjusted to ~7.3 with NaOH. In a subset of cases, a half-sucrose cutting solution (75 mM NaCl) with additional penicillin-streptomycin (1 kU/ml) and nystatin (3 U/ml) was used to accommodate the preferences of a separate group who received the extra slices. Extracellular solutions were continuously aerated with carbogen (95 % O_2_, 5 % CO_2_) without interruption. The internal solution contained (in mM): 130 K-gluconate, 10 HEPES, 4 KCl, 4 NaCl, 15 di-tris-phosphocreatine, 0.3 Na_2_-GTP, 4.0 Mg-ATP, pH adjusted to ~7.3 with KOH, leading to a final [K^+^] of 143 mM. For voltage clamp experiments involving depolarized command potentials, cells were initially patched with the aforementioned potassium-based internal solution for electrophysiological characterization of cell type, then re-patched with the cesium-based internal solution of the following composition (in mM): 120 CsOH, 120 D-gluconate, 4 NaOH, 10 tetraethylammonium (TEA) hydroxide, 10 HEPES, 4 CsCl, 4 QX314 chloride, 10 phosphocreatine di(tris), 0.3 Na_2_-GTP, 4.0 Mg-ATP, pH adjusted to ~7.3 with D-gluconate (~ 20 mM). All materials were obtained from Sigma-Aldrich and Tocris. Liquid junction potential (LJP) measured under identical conditions to the recordings was ~11 mV for the potassium-based internal solution, or ~8 mV for the cesium-based internal solution; membrane potential values reported in this study are without LJP correction, following convention in the field. Excitatory and inhibitory postsynaptic currents (EPSCs and IPSCs) were therefore measured at the respective holding potential of −70 mV or +15 mV to arrive at the intended reversal potentials considering LJP. Patch pipettes (~2–5 MΩ) were pulled from borosilicate glass capillaries (WPI PG52151-4) using a pipette puller (Sutter P-1000). Pipette capacitance was fully neutralized during recordings, and bridge was fully balanced. Series resistance (R_s_) was monitored throughout the experiments, and Rs below 25 MΩ with less than 15 % deviation was accepted.

Neocortical pyramidal neurons (PNs) and interneurons were selected from layer 2/3 (L2/3), at 500–1,500 μm from the cortical surface (typically ~1,000 μm). L2/3 was distinguished by the boundaries with the molecular L1 and the granular L4. Human interneurons were further categorized into fast-spiking interneurons (FSINs) or non-fast-spiking interneurons (nFSINs) by electrophysiology. Distance was measured using a motorized stage calibrated with a micrometer. Input resistance (R_in_) of a cell was calculated from the linear regression of the i-V curve crossing the origin. Sag ratio was calculated from the hyperpolarizing direct current injection with a predefined amplitude (−200 pA, 500 ms). Action potential (AP) detection threshold was 0 mV. AP threshold was defined as V_m_ at dV/dt = 10 (V/s). AP amplitude was defined as the AP peak relative to the AP threshold. AP half-width was defined as the time between the rising and the falling phase of the AP at half AP amplitude from threshold. For stimulation experiments, a stimulus isolator (AMPI Isoflex or WPI A365) was used to deliver monophasic bipolar pulses (pulse width, 0.2 ms) via a concentric bipolar stimulation electrode (FHC 30201). Stimulation intensities above 500 μA were avoided due to water electrolysis. Stimulation electrodes were placed on the slice surface ~250 μm (or at varying distances for Figure 1) laterally from each recorded cell, at the same cortical depth relative to the recorded cell. Distances were measured with a motorized stage calibrated with a stage micrometer.

The rodent experiment component of this work was approved by the Institutional Animal Care and Use Committee (IACUC) at BCH (00001916). Male and female adult mice, ages > 16 weeks, were used for the electrophysiology experiments. Stereotaxic surgery involved the injection of AAV2-syn-DIO-mCherry into the prefrontal cortex (PFC) (AP +2.1, ML ±0.4, DV −1.9 (mm) from bregma) of PV-Cre or Sst-Cre mice (008069, 013044; Jackson Laboratory), respectively, at least 4 weeks prior to slicing. Mice were anesthetized with ~2 % (v/v) isoflurane while being maintained at ~36 °C throughout the procedure. Labeled interneuron subtypes were visualized with a collimated LED (CoolLED pE-300 Ultra) at peak excitation wavelength of ~550 nm. Human and rodent slice experiments were performed under identical conditions.

### Data analysis and statistics

Data were acquired with a MultiClamp 700B amplifier, Digidata 1550B digitizer, and pClamp 11 software (Molecular Devices), sampled at 20 kHz and filtered with a 4^th^-order Bessel filter with cutoff frequency of 4 kHz for voltage clamp experiments or 10 kHz for current clamp experiments. Data were analyzed and visualized with custom codes in MATLAB including PVBS (https://github.com/flosfor/pvbs), and Prism 8 (GraphPad). Data points indicate mean ± S.E.M., unless specified otherwise (in Supplementary Figure 2). Mann-Whitney U test or the Wilcoxon signed-rank test were respectively used to compare unpaired or paired data, unless specified otherwise. Two-way ANOVA was used to compare firing rates between neuronal populations in response to direct current injection. Statistical significance was accepted when *P* < 0.05, denoted by asterisks (^*^P < 0.05, ^**^P < 0.01, ^***^P < 0.001).

## Data Availability

The original contributions presented in the study are included in the article/Supplementary material, further inquiries can be directed to the corresponding authors.
