# Supplementary material for: High-frequency, high-intensity electrical stimulation selectively activates human fast-spiking interneurons
Source: Front Synaptic Neurosci. 2026 Jun 26;18:1843277. doi: 10.3389/fnsyn.2026.1843277 (PMC13352477; doi:10.3389/fnsyn.2026.1843277)
Supplement: Supplementary file 1 [file Data_Sheet_1.pdf]

High-Frequency, High-Intensity Electrical Stimulation Selectively Activates Human Fast-Spiking Interneurons

Jaeyoung Yoon [1,2,*], Ricardo Silva [1,3], Scellig Stone [4,5], Hart Lidov [6,7], Emily Osterweil [2,8], Brielle R. Ferguson [1,9,*]

[1] F. M. Kirby Neurobiology Center, Boston Children's Hospital, Boston, MA 02115, USA.
[2] Department of Neurology, Harvard Medical School, Boston, MA 02115, USA.
[3] Department of Neurobiology, Harvard Medical School, Boston, MA 02115, USA.
[4] Department of Neurosurgery, Boston Children's Hospital, Boston, MA 02115, USA.
[5] Department of Neurosurgery, Harvard Medical School, Boston, MA 02115, USA.
[6] Department of Pathology, Boston Children's Hospital, Boston, MA 02115, USA.
[7] Department of Pathology, Harvard Medical School, Boston, MA 02115, USA.
[8] Zander & Wyss Translational Neuroscience Center, Boston Children's Hospital, Boston, MA 02115, USA.
[9] Department of Genetics, Harvard Medical School, Boston, MA 02115, USA.
[*] Correspondence: jy.yoon@tch.harvard.edu, brielle.ferguson@childrens.harvard.edu

Supplementary materials

Supplementary Table S1

ID	Age	Sex	Area	Hemisphere
01	10	F	PL	R
02	14	F	TL	R
03	1†	F	TL	L
04	1‡	M	FL	L
05	9	F	FL	L
06	3	M	FL	L
07	21	F	TL	R
08	9	F	FL	R
09	10	M	FL	R
10	6	M	PL	L

Abbreviations: M, male; F, female, FL, frontal lobe; TL, temporal lobe; PL, parietal lobe; L, left; R, right. † 13 months. ‡ 19 months.

Supplementary Table S1. Patient and tissue information.

Supplementary Figure S1

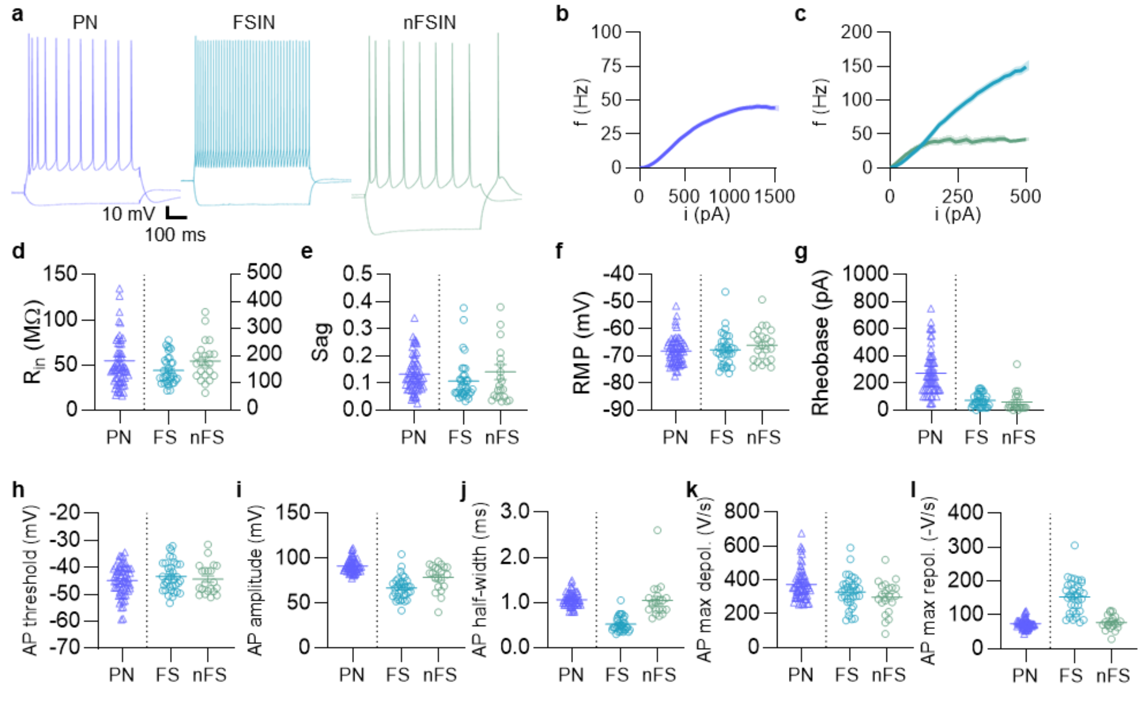

Supplementary Figure S1. Human neuronal intrinsic properties (n = 62, 35, 22 cells, 10 humans; PN, FSIN, nFSIN). **(a)** Representative traces. Traces are from direct current injection at -200 pA and 2 × rheobase. **(b)** PN firing rates. **(c)** FSIN (cyan) and nFSIN (green) firing rates. **(d)** Input resistance. **(e)** Sag ratio. **(f)** RMP. **(g)** Rheobase. **(h)** AP threshold. **(i)** AP amplitude. **(j)** AP half-width. **(k)** AP maximum rate of depolarization (upstroke velocity). **(l)** AP maximum rate of repolarization (downstroke velocity).

Supplementary Figure S2

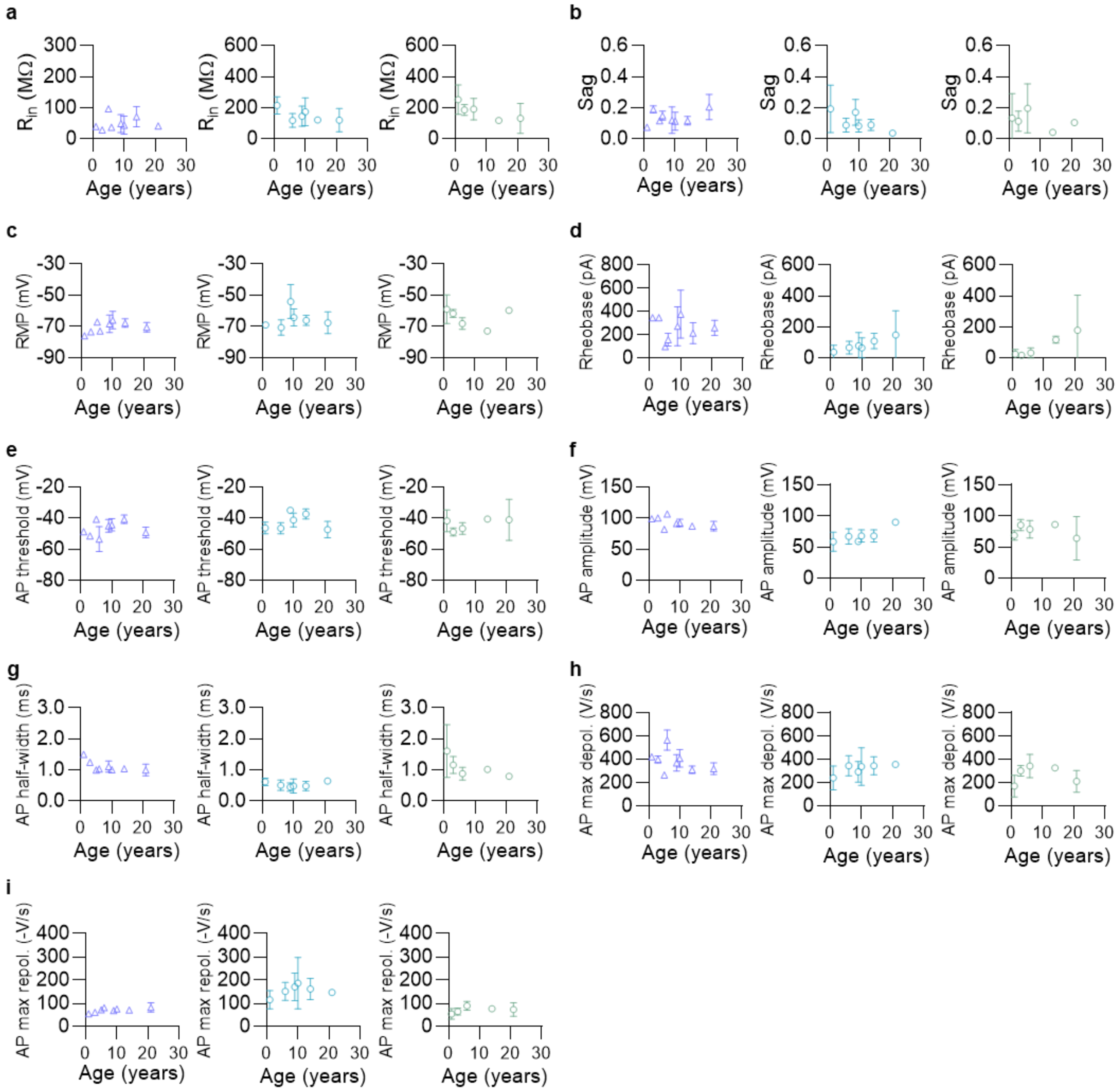

Supplementary Figure S2. Human neuronal intrinsic properties by age. Note that data points indicate mean ± S.D., instead of mean ± S.E.M., as the latter were too small to be visualized. Data are from Supplementary Fig. S1. **(a)** Input resistances of PNs (left; n = 1, 2, 1, 5, 12, 17, 15, 9, from ages 1, 3, 5, 6, 9, 10, 14, 21 (y), respectively), FSINs (middle; n = 5, 17, 1, 3, 7, 2, from ages 1, 6, 9, 10, 14, 21 (y), respectively), and nFSINs (right; n = 3, 3, 10, 4, 2, from ages 1, 3, 6, 14, 21 (y), respectively). **(b)** Sag ratio. The number of cells in each age are identical across all panels. **(c)** RMP. **(d)** Rheobase. **(e)** AP threshold. **(f)** AP amplitude. **(g)** AP half-width. **(h)** AP maximum rate of depolarization (upstroke velocity). **(i)** AP maximum rate of repolarization (downstroke velocity).

Supplementary Figure S3

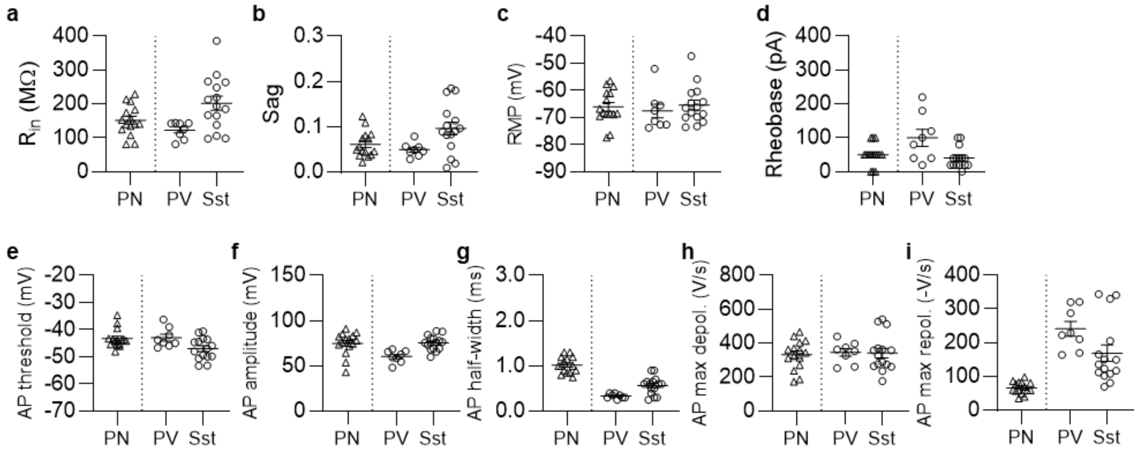

Supplementary Figure S3. Mouse neuronal intrinsic properties (n = 15, 8, 15; PN, PVIN, SstIN). **(a)** Input resistance. **(b)** Sag ratio. **(c)** RMP. **(d)** Rheobase. **(e)** AP threshold. **(f)** AP amplitude. **(g)** AP half-width. **(h)** AP maximum rate of depolarization (upstroke velocity). **(i)** AP maximum rate of repolarization (downstroke velocity).

Supplementary Figure S4

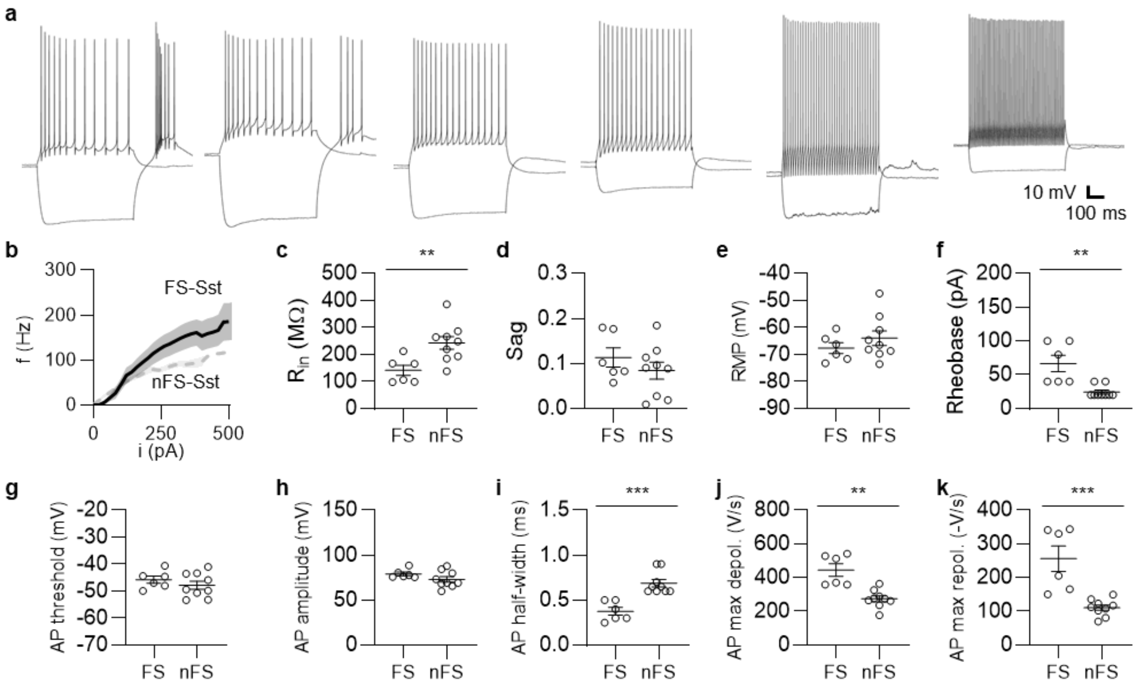

Supplementary Figure S4. Mouse SstINs are electrophysiologically heterogeneous. **(a)** Representative traces from virally labeled SstINs displaying diverse electrophysiological properties. Traces are from direct current injection at -200 pA and 2 × rheobase. **(b)** Firing rates of the fast-spiking SstINs (FS-Sst; n = 6) and non-fast-spiking SstINs (nFS-Sst; n = 9). FS-Sst and nFS-Sst were distinguished by AP half-width at 0.5 ms. **(c)** Input resistance (P = 0.0076, Mann-Whitney U test). **(d)** Sag ratio. **(e)** RMP. **(f)** Rheobase (P = 0.0034). **(g)** AP threshold. **(h)** AP amplitude. **(i)** AP half-width (P = 0.0002). **(j)** AP maximum rate of depolarization (upstroke velocity) (P = 0.0016). **(k)** AP maximum rate of repolarization (downstroke velocity) (P = 0.0004).

Supplementary Figure S5

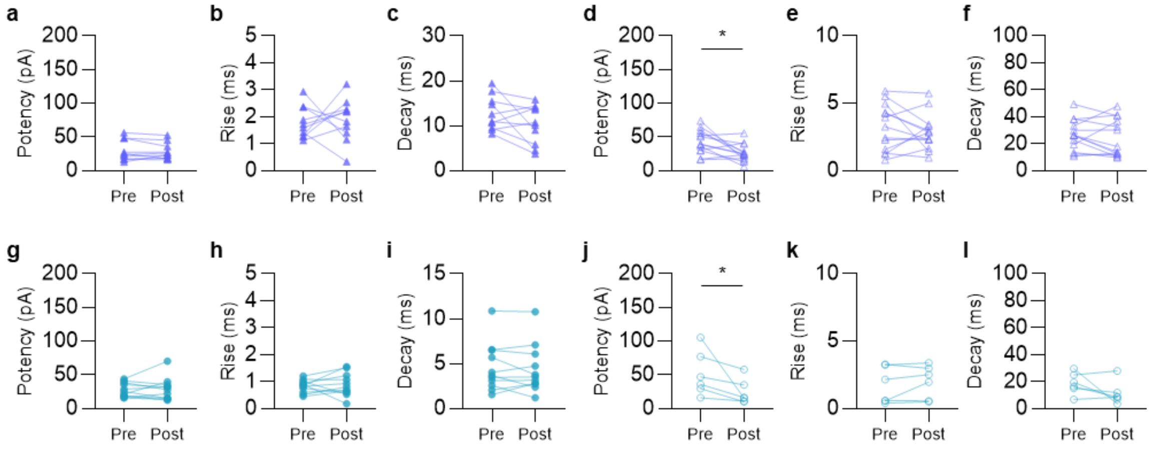

Supplementary Figure S5. Minimally evoked EPSC and IPSC kinetics were not affected by high-frequency, high-intensity stimulation. Rise and decay times were calculated as the time between 10 % and 90 % of peak, respectively from the rising phase and the decay phase of the postsynaptic response. **(a)** Potency (failure-excluded means) of EPSCs at postsynaptic PNs was not changed after the stimulation trains (n = 10, 3 humans; P = 0.5566, Wilcoxon signed-rank test). **(b)** Rise time of EPSCs at PNs (P = 0.9219). **(c)** Decay time of EPSCs at PNs (P = 0.1055). **(d)** Potency of IPSCs at PNs was decreased after the stimulation trains (n = 13 cells, 1 human; P = 0.0105). **(e)** Rise time of IPSCs at PNs (P = 0.3757). **(f)** Decay time of IPSCs at PNs (P = 0.4143). **(g)** Potency of EPSCs at FSINs was not affected by the stimulation trains (n = 11 cells, 3 humans; P = 0.7002). **(h)** Rise time of EPSCs at FSINs (P = 0.5195). **(i)** Decay time of EPSCs at FSINs (P = 0.7646). **(j)** Potency of IPSCs at FSINs was decreased after the stimulation trains (n = 6 cells, 1 human; P = 0.0313). **(g)** Rise time of IPSCs at FSINs (P = 0.3125). **(h)** Decay time of IPSCs at FSINs (P = 0.1563).
